# Clinical Profile and Outcome of Patients With Advanced Hepatocellular Carcinoma (HCC) Taking Sorafenib or Lenvatinib: Real-World Experience From a Low-Middle Resource Country

**DOI:** 10.7759/cureus.83681

**Published:** 2025-05-07

**Authors:** Kinza Sabir, Jawayria Sajid, Nauman I Butt, Barak Waris, Amal Zahra, Muhammad Shoaib

**Affiliations:** 1 Medical Oncology, Nawaz Sharif Social Security Teaching Hospital, Lahore, PAK; 2 Medical Oncology, Shalamar Hospital, Shalamar Medical and Dental College, Lahore, PAK; 3 Internal Medicine, Chaudhry Muhammad Akram Teaching and Research Hospital, Azra Naheed Medical College, Superior University, Lahore, PAK

**Keywords:** barcelona clinic liver cancer (bclc) staging, child-pugh scoring system, chronic hepatitis virus c infection, eastern cooperative oncology group (ecog) performance status, hand-foot syndrome, hepatocellular carcinoma (hcc), lenvatinib, sorafenib

## Abstract

Background and objective

Hepatocellular carcinoma (HCC) incidence is high in Asian countries, and the age-standardized ratio in Pakistan is 7.6/100000 for males and 2.8/100000 for females, where patients often present with advanced-stage disease due to limited early detection. While the tyrosine kinase inhibitor sorafenib has been the standard first-line therapy, another tyrosine kinase inhibitor, lenvatinib, has recently gained attention as a potential alternative. However, local data comparing their effectiveness and safety is scarce. The aim of the study was to assess the clinical profile of patients with advanced HCC and treatment outcomes with sorafenib or lenvatinib in a tertiary care setting in Pakistan.

Methods

This retrospective, data-based study was conducted at the Department of Medical Oncology, Nawaz Sharif Social Security Teaching Hospital, Lahore, Pakistan. Medical records of 104 patients classified as having Barcelona clinic liver cancer (BCLC) stage C HCC were included. Patients with incomplete medical records, missing essential clinical data, those lost to follow-up, or who died before initiation of treatment were excluded from the analysis. Demographic information, including age, gender, hepatitis B and C status, and co-morbid conditions, was evaluated. For documentation of baseline parameters, Eastern Cooperative Oncology Group (ECOG) performance status, number of lesions on CT scan, evidence of invasive or metastatic disease, and liver function parameters categorized by Child-Pugh class (A to C) were assessed. Treatment records were examined to determine whether patients received Sorafenib or Lenvatinib, and the outcome and adverse effects were noted. Results of follow-up CT scan performed at six months were used as the primary outcome measure to assess disease status, categorized as complete resolution, partial resolution, or no response/progression. Data were entered and analyzed using SPSS version 23 (IBM Inc., Armonk, New York).

Results

The mean age of the patients was 64.9±8.3 years, with 74 (71.2%) males. All patients had advanced HCC at BCLC stage C. Chronic hepatitis C was reported in 92 (88.5%) patients, while chronic hepatitis B in 02 (1.9%). The most frequent co-morbid conditions were hypertension (26, 25.0%) and diabetes mellitus (18, 17.3%). The majority of the patients (40, 38.5%) had grade 2 ECOG performance status, with 68 (65.4%) having multifocal HCC and 62 (59.6%) vascular invasion. Metastases were seen in 38 (36.5%) patients, with nodal metastasis being the most frequent site (18, 17.3%). The mean Child-Pugh score was 6.5±1.3, with the majority of the patients (62, 59.6%) having Child-Pugh class A. With regards to treatment, 66 (63.5%) patients received sorafenib while 38 (36.5%) received lenvatinib. At the six-month follow-up, repeat CT scan demonstrated complete resolution of HCC in 04 (3.8%) patients and partial resolution in 58 (55.8%) patients, whereas 42 (40.4%) patients showed no response or progression. Hand-foot syndrome was the most frequent adverse effect seen in eight (7.7%) patients, followed by diarrhea in four (3.8%) patients, and mucositis in two (1.9%) patients.

Conclusion

In this study, sorafenib and lenvatinib were both found to be effective treatment options for patients with advanced HCC at BCLC stage C, with partial resolution observed in the majority of patients.

## Introduction

Hepatocellular carcinoma (HCC) is the most prevalent type of primary liver cancer, responsible for over 90% of such cases [1]. Globally, HCC ranks as the fifth most frequently diagnosed cancer, and is the second leading cause of cancer-related deaths in men, following lung cancer [2,3]. The overall five-year survival rate remains low at approximately 18%, making it one of the most lethal forms of cancer after pancreatic cancer [3,4]. More than 80% of newly diagnosed hepatocellular carcinoma cases occur in low- and middle-resource countries, especially in regions like Sub-Saharan Africa, South-East Asia, South Asia, including Pakistan, where hepatitis C and B virus infections remain widespread [5].

HCC most commonly develops in individuals with underlying liver conditions, particularly cirrhosis, which is present in about 80-90% of those diagnosed with it [4]. Among patients with cirrhosis, the likelihood of developing HCC ranges from 2% to 4% annually. Key risk factors contributing to the development of HCC include chronic viral infections with hepatitis B (HBV) or hepatitis C (HCV) in over 70% cases, excessive alcohol consumption leading to liver damage and liver disease related to obesity such as non-alcoholic fatty liver disease (NAFLD) and its more severe form, non-alcoholic steatohepatitis (NASH) [1,2]. In HBV infection, cancer development is mainly driven by the insertion of viral genetic material into the host's DNA where as for HCV, ongoing liver inflammation leads to tissue scarring, cell injury, and repeated cycles of repair, all of which play a central role in the emergence of liver cancer [6].

HCC is a leading cause of cancer-related mortality, particularly in resource-limited countries like Pakistan, where late-stage presentation is common due to limited screening and early detection. With most patients diagnosed at an advanced stage, systemic therapy becomes the mainstay of treatment [7,8]. A tyrosine kinase inhibitor, sorafenib, has long been the standard first-line therapy; however, another tyrosine kinase inhibitor, lenvatinib, has recently emerged as an alternative with comparable efficacy and potentially differing toxicity profiles [7,8]. Despite global data, there is limited local evidence comparing these treatments in real-world clinical settings in Pakistan. This study aims to evaluate the clinical characteristics and treatment outcomes of advanced HCC patients at Barcelona Clinic Liver Cancer (BCLC) stage C treated with sorafenib or lenvatinib in a local tertiary care setting, thereby contributing valuable data to guide clinical decisions and optimize patient care in resource-limited environments.

## Materials and methods

This retrospective, data-based study was conducted at the Department of Medical Oncology, Nawaz Sharif Social Security Teaching Hospital, Lahore, Pakistan, to evaluate the clinical profile and treatment outcomes of patients with advanced HCC treated with sorafenib or lenvatinib. Medical records of patients classified as having BCLC stage C HCC were included. Patients with incomplete medical records, missing essential clinical data, those lost to follow-up, or who died before initiation of treatment were excluded from the analysis. The study included medical records from January 2024 to December 2024, and a total of 104 patient files were included after obtaining approval from the institutional ethics committee. All patient data were handled in accordance with institutional and ethical guidelines to ensure confidentiality. Identifiable information was removed, and records were anonymized prior to analysis to maintain patient privacy. Access to data was restricted to authorized study personnel only.

All patients had BCLC stage C HCC. Demographic information, including age, gender, hepatitis B and C status, and co-morbid conditions, was evaluated. For documentation of baseline parameters, the medical records were further reviewed for Eastern Cooperative Oncology Group (ECOG) performance status, number of lesions on CT scan, evidence of invasive or metastatic disease, and liver function parameters categorized by Child-Pugh class (A to C). Radiologic or clinical documentation of vascular invasion, lymph node involvement, and distant metastases was also assessed. Treatment records were examined to determine whether patients received sorafenib or lenvatinib. Treatment outcomes and adverse effects were subsequently evaluated. Results of follow-up CT scans performed at six months were used as the primary outcome measure to assess disease status, categorized as complete resolution, partial resolution, or no response/progression.

Data were entered and analyzed using SPSS version 23 (IBM Inc., Armonk, New York). Descriptive statistics were used to summarize baseline characteristics. Post-stratification, the Chi-squared test and Fisher's exact test were applied to assess associations between outcome, adverse effects, and treatment given, with a p-value of ≤0.05 considered statistically significant.

## Results

Medical records of 104 patients with advanced HCC (BCLC stage C) were included in the study. The mean age of the participants was 64.9±8.3 years, with 74 (71.2%) males and 30 (28.8%) females. Chronic hepatitis C was reported in 92 (88.5%) patients, while chronic hepatitis B was reported in two (1.9%). The most frequent co-morbid conditions were hypertension (26, 25.0%) and diabetes mellitus (18, 17.3%) as depicted in Table 1. The majority of the patients (40, 38.5%) had grade 2 ECOG performance status, with 68 (65.4%) having multifocal HCC and 62 (59.6%) vascular invasion. Metastases were seen in 38 (36.5%) patients, with nodal metastasis being the most frequent site (18, 17.3%) as reported in Table 1.

**Table 1:** Clinical profile of the patients (N=104) COPD - chronic obstructive pulmonary disease; DVT - deep venous thrombosis; ​​​​​​​ECOG - Eastern Cooperative Oncology Group

Clinical parameter	Result n (%)
Co-morbid conditions	
Hypertension	26 (25.0%)
Diabetes mellitus	18 (17.3%)
COPD	8 (7.7%)
Ischemic heart disease	6 (5.8%)
Cholelithiasis	2 (1.9%)
Lower limb DVT	2 (1.9%)
ECOG performance status	
Grade 0	26 (25.0%)
Grade 1	24 (23.1%)
Grade 2	40 (38.5%)
Grade 3	14 (13.5%)
Number of lesions	
Unifocal	36 (34.6%)
Multifocal	68 (65.4%)
Vascular invasion	
Present	62 (59.6%)
Absent	42 (40.4%)
Site of metastasis	
Nodal	18 (17.3%)
Lung	10 (9.6%)
Bone	8 (7.7%)
Adrenal	2 (1.9%)
No metastasis	66 (63.5%)

Mean serum bilirubin was 0.8±0.5 mg/dl, with 90 (86.5%) patients having serum bilirubin <2 mg/dl, as shown in Table 2. Four (3.8%) patients had severe refractory ascites, while 32 (30.8%) had mild-to-moderate ascites. Mean serum albumin was 3.3±0.5 g/dl, and 56 (53.8%) patients had serum albumin between 2.8-3.5 g/dl. The mean international normalized ratio (INR) was 1.2±0.4, and 88 (84.6%) patients had INR <1.7. Hepatic encephalopathy was not seen in any patient. The mean Child-Pugh score was 6.5±1.3, with the majority of the patients (62, 59.6%) having Child-Pugh class A as demonstrated in Table 2. Mean serum alpha-fetoprotein (AFP) and protein-induced by vitamin K absence or antagonist-II (PIVKA-II) levels were 168.2±259.0 ng/ml and 63,328.8±177,862.7 mAU/ml, respectively.

**Table 2:** Child-Pugh score and its parameters (N=104) INR - international normalized ratio

Clinical parameter	Result, n (%)
Child-Pugh category	
Child's A (Score 5-6)	62 (59.6%)
Child's B (Score 7-9)	36 (34.6%)
Child's C (Score 10-15)	06 (5.8%)
Serum bilirubin	
<2 mg/dl	90 (86.5%)
2-3 mg/dl	10 (9.6%)
>3 mg/dl	04 (3.8%)
Ascites	
None	68 (65.4%)
Mild-to-moderate	32 (30.8%)
Severe	04 (2.8%)
Serum albumin	
>3.5 g/dl	36 (34.6%)
3.5-2.8 g/dl	56 (53.8%)
<2.8 g/dl	12 (11.5%)
INR	
<1.7	88 (84.6%)
1.7-2.3	12 (11.5%)
>2.3	04 (3.8%)
Hepatic encephalopathy	
None	104 (100.0%)

With regard to treatment, 66 (63.5%) patients received sorafenib while 38 (36.5%) received lenvatinib. The distribution of clinical parameters of the patients with regard to drugs received is shown in Table 3. The lenvatinib group (n=38) had a higher proportion of males (32, 84.2%) compared to sorafenib (42, 63.6%), and more patients with better performance status (ECOG 0-1: 24, 63.2% vs. 26, 39.4%) and better liver function (Child-Pugh A: 26, 68.4% vs. 36, 54.5%). Bone (8, 12.1%) and adrenal (2, 3.0%) metastases occurred only in the sorafenib group. Both groups had similar rates of multifocal disease (24, 63.2% vs. 44, 66.7%) and vascular invasion (24, 63.2% vs. 38, 57.6%). Nodal metastases were more frequent with lenvatinib (10, 26.3%) compared to sorafenib (8, 12.1%).

**Table 3:** Clinical profile of the patients according to drug received (N=104) COPD - chronic obstructive pulmonary disease; ​​​​​​DVT - deep venous thrombosis; ECOG: Eastern Cooperative Oncology Group

Clinical parameter	Sorafenib (n=66)	Lenvatinib (n=38)
Gender		
Female	24 (36.4%)	6 (15.8%)
Male	42 (63.6%)	32 (84.2%)
Co-morbid conditions		
Hypertension	16 (26.3%)	10 (26.3%)
Diabetes mellitus	6 (9.1%)	12 (31.6%)
COPD	8 (12.1%)	0 (0.0%)
Ischemic heart disease	2 (3.0%)	4 (10.5%)
Cholelithiasis	2 (3.0%)	0 (0.0%)
Lower limb DVT	0 (0.0%)	2 (5.3%)
ECOG performance status		
Grade 0	14 (21.2%)	12 (31.6%)
Grade 1	12 (18.2%)	12 (31.6%)
Grade 2	32 (48.5%)	8 (21.1%)
Grade 3	08 (12.1%)	6 (15.8%)
Number of lesions		
Unifocal	22 (33.3%)	14 (36.8%)
Multifocal	44 (66.7%)	24 (63.2%)
Vascular invasion		
Present	38 (57.6%)	24 (63.2%)
Absent	28 (42.4%)	14 (36.8%)
Site of metastasis		
Nodal	8 (12.1%)	10 (26.3%)
Lung	6 (9.1%)	4 (10.5%)
Bone	8 (12.1%)	0 (0.0%)
Adrenal	2 (3.0%)	0 (0.0%)
No metastasis	42 (63.7%)	28 (63.2%)
Child-Pugh category		
Child's A	36 (54.5%)	26 (68.4%)
Child's B	26 (39.4%)	10 (26.3%)
Child's C	04 (6.1%)	02 (5.3%)

At the six-month follow-up, repeat CT scan demonstrated complete resolution of HCC in four (3.8%) patients and partial resolution in 58 (55.8%) patients, whereas 42 (40.4%) patients showed no response or progression. Hand-foot syndrome was the most frequent adverse effect seen in eight (7.7%) patients, followed by diarrhea in four(3.8%) patients, and mucositis in two (1.9%) patients. The stratification of outcomes and adverse effects with regard to medication received is reported in Table 4.

**Table 4:** Outcome and adverse effects according to the medication received (N=104) *p-value ≤0.05 significant using Chi-squared test and Fisher's exact test

Parameters	Sorafenib (n=66)	Lenvatinib (n=38)	p-value*	Pearson Chi-squared value
Outcome			0.075	5.171
Complete resolution	4 (100.0%)	0 (0.0%)		
Partial resolution	32 (55.2%)	26 (44.8%)		
No response/progression	30 (71.4%)	12 (28.6%)		
Adverse effects				
Hand-foot syndrome	8 (100.0%)	0 (0.0%)	0.026*	4.990
Mucositis	2 (100.0%)	0 (0.0%)	0.532	1.174
Diarrhea	2 (50.0%)	2 (50.0%)	0.622	0.325

## Discussion

Curative treatments for early-stage HCC, such as surgery, liver transplantation, and ablation, can enhance survival [9,10]. However, most HCC cases are diagnosed at an advanced stage, often in patients with chronic liver disease, limiting the use of these curative approaches [10,11]. For advanced HCC, systemic therapies like sorafenib have been the primary treatment, improving both survival and quality of life [12,13]. Recent studies have shown lenvatinib, a multikinase inhibitor, to be as effective as sorafenib in overall survival and offered better progression-free survival and response rates [14,15].

In the present study with 104 patients of BCLC stage C HCC, at the six-month follow-up, complete resolution of HCC was observed in 6.1% of patients receiving sorafenib, whereas no patients in the lenvatinib group achieved complete resolution. Partial resolution was seen in 48.5% of patients treated with sorafenib and 68.4% of patients receiving lenvatinib. In contrast, 45.5% of patients receiving sorafenib and 31.6% receiving lenvatinib showed no response or disease progression. This difference in treatment outcomes between the sorafenib and lenvatinib groups, specifically the higher complete resolution rate seen with sorafenib (6.1%) and the higher partial resolution rate with lenvatinib (68.4%), may be influenced by multiple factors beyond the difference in sample size (66 vs. 38). While a smaller group size in the lenvatinib group could reduce the likelihood of observing rare events like complete resolution, this alone does not fully explain the outcome pattern. Although all patients in this study were classified as having advanced hepatocellular carcinoma corresponding to BCLC stage C, there was notable clinical heterogeneity between the sorafenib and lenvatinib groups. Notably, patients in the lenvatinib group had better baseline liver function (Child-Pugh A in 68.4% vs. 54.5%) and performance status (ECOG 0-1 in 63.2% vs. 39.4%), which are typically associated with improved treatment tolerance and response. However, complete resolution was only observed in the sorafenib group, possibly due to individual tumor biology or exceptional drug response in a few patients. Additionally, differences in metastatic patterns and comorbidities, along with real-world treatment variables such as adherence, dose modifications, and supportive care, could have also contributed to these variations. These differences, although subtle, may have influenced treatment outcomes and response patterns within the study, despite all patients being within the same BCLC stage.

In our study, sorafenib was associated with a higher rate of complete resolution of BCLC stage C HCC compared to lenvatinib, but it was also linked to a greater incidence of adverse effects, particularly hand-foot syndrome. In the present study, hand-foot syndrome was the most commonly reported adverse effect, occurring in 7.7% of patients, all of whom received sorafenib. Diarrhea was reported in 3.8%, evenly distributed between both groups, while mucositis was seen in 1.9%, also among sorafenib users. This observation contrasts with some real-world data, where lenvatinib has shown comparable efficacy to sorafenib in terms of progression-free survival and overall survival, without a significant difference in the incidence of hand-foot syndrome. A large multi-center study, such as the REFLECT trial, demonstrated that lenvatinib was non-inferior to sorafenib in terms of overall survival for patients with advanced HCC, and the incidence of hand-foot syndrome was similar between the two treatment groups [14]. The study indicated that while lenvatinib might offer similar efficacy in controlling disease progression, it could potentially have a different toxicity profile, with adverse effects such as hypertension and diarrhea being more prominent in the lenvatinib group, and hand-foot syndrome being less common [14]. In the study by Naqi et al., among the 18 Pakistani patients of advanced HCC, eight experienced stable disease, two showed partial response, and eight had disease progression, while no patients achieved a complete response, and hand-foot skin reaction was seen in 33% [16]. Masood et al. reported diarrhea in 40% and hand-foot syndrome in 36.7% of HCC patients taking sorafenib from Rawalpindi, Pakistan [17]. Our study's results may reflect the unique characteristics of the local population or differences in clinical practice. The relatively higher incidence of hand-foot syndrome in the sorafenib group in our study could be attributed to the small sample size, differences in patient characteristics, or underreporting of less severe adverse events with lenvatinib due to a more limited follow-up period.

This study has several limitations that should be considered when interpreting the results. Firstly, the sample size of 104 patients is relatively small, which may limit the generalizability of the findings to larger populations. Secondly, the study was conducted at a single institution, which could introduce bias related to local treatment practices and patient demographics. Additionally, as a retrospective study, it is subject to the inherent limitations of using pre-existing data, such as incomplete or missing records, which may affect the accuracy of some clinical assessments. Finally, the relatively short follow-up period of six months may not fully capture the long-term outcomes and potential late adverse effects of the treatments studied. This underscores the importance of context when interpreting clinical outcomes from real-world data, as regional factors such as healthcare infrastructure, access to medication, and patient management practices can significantly influence both treatment outcomes and adverse effect reporting.

Based on the findings and limitations of this study, several recommendations can be made for future research. Given that our study was retrospective, data collection was based on existing medical records, which may have resulted in variations in how clinical parameters, outcomes, and adverse effects were documented and managed. Real-world data from larger, multi-center trials with larger sample size could offer a broader understanding of how these drugs perform across diverse patient populations and healthcare settings, potentially explaining the differences in adverse event profiles between our study and others, further confirming the efficacy and safety of sorafenib and lenvatinib in the treatment of advanced HCC at BCLC stage C and to enhance the generalizability of the results. Additionally, longer follow-up periods are needed to better assess long-term treatment outcomes, survival rates, and the persistence of adverse effects. Future studies should also consider incorporating more diverse patient populations to explore potential differences in treatment response across varying demographic and clinical profiles. Finally, prospective studies are recommended to validate the findings and reduce the inherent biases of retrospective data collection.

## Conclusions

In this study, sorafenib and lenvatinib were both found to be effective treatment options for patients with advanced HCC at BCLC stage C, with partial resolution observed in the majority of patients. Sorafenib, however, was associated with a higher rate of complete resolution and a greater incidence of adverse effects, particularly hand-foot syndrome. The results highlight the importance of personalized treatment strategies based on patient characteristics and the potential for optimizing treatment regimens in the management of advanced HCC at BCLC stage C. Further studies with larger sample sizes and longer follow-up periods are needed to better assess long-term outcomes and the comparative efficacy of these therapies in diverse patient populations.
